# Diabetic foot ulcer mobile detection system using smart phone thermal camera: a feasibility study

**DOI:** 10.1186/s12938-017-0408-x

**Published:** 2017-10-03

**Authors:** Luay Fraiwan, Mohanad AlKhodari, Jolu Ninan, Basil Mustafa, Adel Saleh, Mohammed Ghazal

**Affiliations:** 1 Electrical and Computer Engineering Deparment, Abu Dhabi University, 59911 Abu Dhabi, United Arab Emirates; 2 Biomedical Engineering Department, Jordan University of Science and Technology, Irbid, 22110 Jordan

**Keywords:** Ulcer detection, Mobile thermal camera, Thermal imaging, Otsu thresholding, Image registration

## Abstract

**Background:**

Nowadays, the whole world is being concerned with a major health problem, which is diabetes. A very common symptom of diabetes is the diabetic foot ulcer (DFU). The early detection of such foot complications can protect diabetic patients from any dangerous stages that develop later and may require foot amputation. This work aims at building a mobile thermal imaging system that can be used as an indicator for possible developing ulcers.

**Methods:**

The proposed system consists of a thermal camera connected to a Samsung smart phone, which is used to acquire thermal images. This thermal imaging system has a simulated temperature gradient of more than 2.2 °C, which represents the temperature difference (in the literature) than can indicate a possible development of ulcers. The acquired images are processed and segmented using basic image processing techniques. The analysis and interpretation is conducted using two techniques: Otsu thresholding technique and Point-to-Point mean difference technique.

**Results:**

The proposed system was implemented under MATLAB Mobile platform and thermal images were analyzed and interpreted. Four testing images (feet images) were used to test this procedure; one image with any temperature variation to the feet, and three images with skin temperature increased to more than 2.2 °C introduced at different locations. With the two techniques applied during the analysis and interpretation stage, the system was successful in identifying the location of the temperature increase.

**Conclusion:**

This work successfully implemented a mobile thermal imaging system that includes an automated method to identify possible ulcers in diabetic patients. This may give diabetic patients the ability for a frequent self-check of possible ulcers. Although this work was implemented in simulated conditions, it provides the necessary feasibility to be further developed and tested in a clinical environment.

## Background

Diabetes Mellitus (DM) is a metabolic chronic disease that is associated with abnormal glucose levels in the blood. There are two causes of Diabetes Mellitus, the first one is the abnormal production of insulin by the pancreas (Type I), while the second cause is related to inadequate cells action to insulin (Type II). Both types of Diabetes Mellitus can pose a serious threat to patients’ health concerning the cardiovascular system, kidneys, and extremities such as the feet [1]. According to the World Health Organization (WHO), this disease has been dramatically spreading and growing worldwide with an estimation of 422 million adults who live with diabetes in 2014, compared to 108 million adults in 1980 [2]. One of the most dangerous symptoms of this disease is foot complications. Around 15 to 25% of diabetic patients are going to suffer from foot complications at a later stage of the disease [3]. These complications occur as a consequence of infection, peripheral ischemia, and ulceration in the foot [4, 5]. Foot ulcer happens mainly because diabetes introduces peripheral neuropathy, which affects the ability of the foot to feel and sense. That being considered, any injury in the foot can go unnoticed [6, 7]. Pre-signs for such complications include fissures, blisters, abundant callus formation, redness, and increased temperature regions [7]. A physician can check and analyze these physical features in order to diagnose the case. Foot complications can severely develop and result in limb amputation within the foot or even death if left untreated (diabetic foot) [8]. In patients with Diabetes Mellitus disease, approximately 85% of all lower extremity amputations are preceded by foot ulcer [5].

Diabetic foot ulcer can be avoided or delayed if adequately treated at an early stage. Currently, the assessment of such foot complications is done frequently by clinicians through analyzing blood circulations, plantar foot pressure, and foot neuropathy [9, 10]. Moreover, specialist clinicians usually assess lower extremity vascular status using Doppler ultrasound. This allows the possibility of getting accurate analysis regarding the current situation of foot ulcers and its risks [11]. However, patients are forced to go for frequent visits to doctors for diabetic foot assessment, which is considered intrusive and costly. In addition, self-assessment is considered difficult because it depends on the knowledge of patients with this disease, and on the usage of medical equipment. The treatments for such complications are commonly associated with therapeutic footwear, foot education, and normal foot care [12]. For example, a modified walking apparatus is used to provide consistent pressure relief at the diabetic patient’s foot. Thus, the prevention of more developed stages of current foot complications situation can be maintained and even healed [13].

The occurrence of diabetic foot complications is often related to the plantar region temperature distribution. Increased temperature may be present in the foot a week before a neuropathic ulcer appears [14]. Researchers often use technologies such as the liquid crystal thermography (LCT) and infrared (IR) thermography to demonstrate the temperature variations [15]. LCT is a color representation proportional to the temperature of the in-contact foot surface with the thermochromic liquid crystal [16]. However, the infrared (IR) thermal imaging is much preferable because of being a non-invasive technology that acquires thermal images based on the heat emitted from the body. Infrared radiations are waves from the electromagnetic spectrum with a range of 760 nm to 1 mm [17]. This technology has made it possible to measure any increased temperature that occurs in some regions within the foot. A 1 °C temperature increase within the foot over the normal foot mean temperature requires an accurate assessment in order to decide whether it is a normal increase or an occurrence of foot ulcers [12, 18, 19]. Moreover, temperature differences of more than 2.2 °C between a region on one foot and the same region on the contra-lateral foot are considered Hyperthermia [14, 19]. Monitoring such differences through thermal images proved to be an efficient way of detecting diabetic foot ulceration.

The aim of this work is to build an ulcer detection/indication system based on a mobile thermal camera and a mobile application. The proposed system would serve as a self-monitoring tool with a mobile app giving diabetic patients the ability to self-check their extremities for any possible ulcer, without the need for frequent visits to the diabetic clinic. The proposed system was implemented using a mobile application where thermal images were acquired, processed, and analyzed for any possible ulceration. Two image-processing techniques were deployed to detect possible ulcers automatically: the Otsu thresholding techniques and the point-to-point difference techniques. Both techniques were tested on thermal images. The implementation of the image processing algorithms was done using MATLAB mobile (Mathworks, Inc.). It was also complied into Java, and a mobile application was built for this purpose.

## Methods

The proposed system consists of a hardware part, which is mainly a mobile thermal image acquisition camera and a smart phone, along with image processing and analysis software. The entire software was run on MATLAB and was later implemented on a mobile phone through MATLAB Mobile Android application. After that, the entire software was compiled into Java code to build a complete and integrated application with user interface.

### Thermal image acquisition system

#### Thermal imaging system

**Fig. 1:**
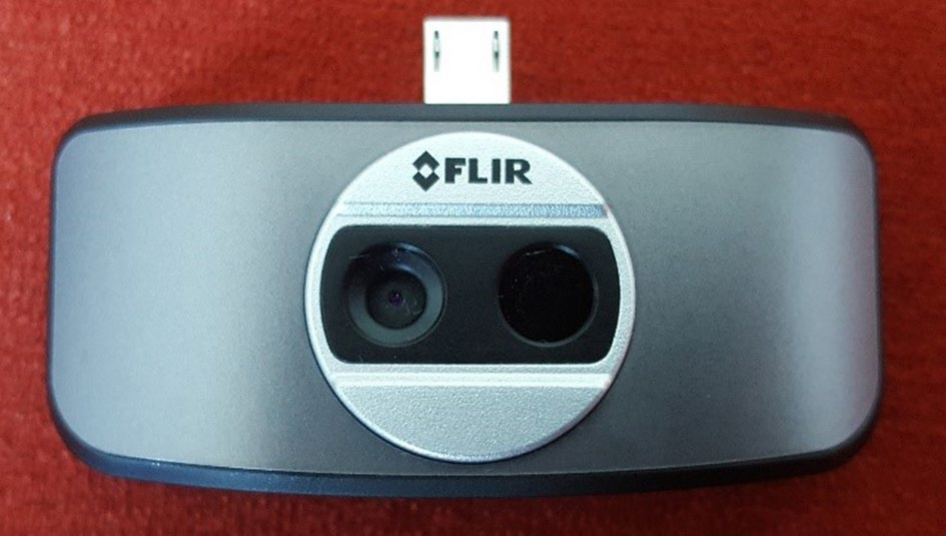
FLIR ONE Infrared Thermal Camera

The thermal image acquisition system, a Smartphone-based system, consists of an infrared (IR) thermal camera, the FLIR ONE (FLIR Systems), shown in Fig. 1. This camera was connected and running on Samsung Galaxy S6 Edge Plus smart phone._bookmark0 FLIR ONE consists of two cameras; Lepton camera, which has a compact long-wave infrared sensor for acquiring thermal images, and a standard camera that provides standard physical details to the raw thermal image. Considering the specifications of this camera, few parameters should be taken into account before applying further analysis on images: scene temperature range, sensitivity, resolution, and emissivity. As shown in Table 1, this IR camera detects temperatures that fall in the range of −20 to 120 °C, with sensitivity in detection of 0.1 °C. FLIR ONE images resolution is not considered the best compared to high end FLIR devices designed for the same purpose. However, as a Smartphone thermal camera, it does provide good images with 160 × 120 resolution [20].

**Fig. 2:**
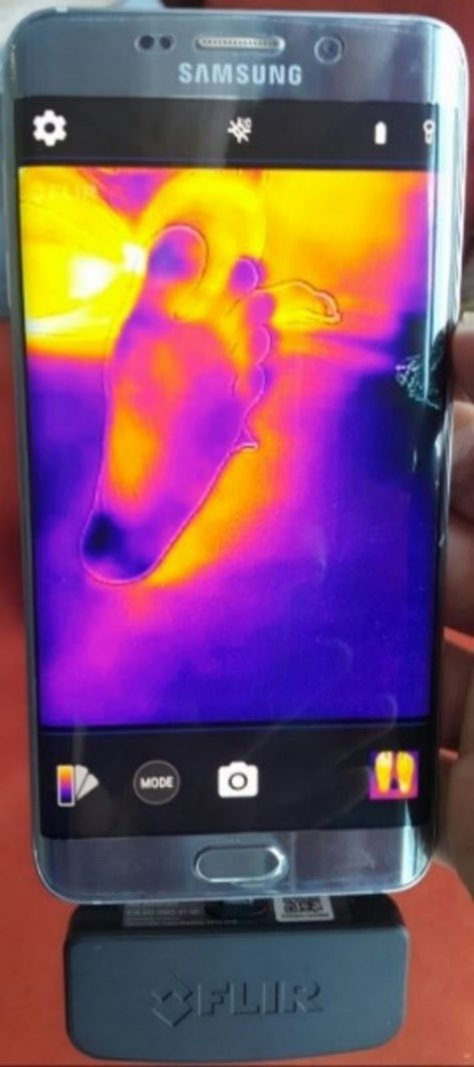
The complete smartphone system along with FLIR ONE app

Regarding the emissivity of objects, FLIR ONE cameras automatically calibrate the scene temperature in order to provide the highest possible accuracy. In this regard, the camera calculates the emissivity of objects, the distance, and the reflected temperature, and then it returns the actual temperature of the object. The accuracy of the FLIR ONE camera and the calibration process have been tested by researchers through placing a reference object with a known temperature and emissivity, such as a blackbody device (Omega BB701), which has 1.0 emissivity and a pre-assigned temperature, in the field-of-view of the camera while conducting a human subject test. Thus, the actual temperature measurements from the camera could be compared with much more accurate devices such as FLIR E60 and IR thermometer (Omega OSXL450) before and after manual calibration procedures. The results showed that both the FLIR E60 and Omega OSXL450 IR thermometer measurements were close to each other and were improved by the calibration process, while FLIR ONE measurements remained the same. FLIR ONE accuracy resulted in having ≈ 2 °C more temperature compared to the other accurate devices while observing the measurements [21], meaning that it was with around ± 5% accuracy in temperature readings. This can be considered a good background for our proposed techniques and analysis as a smart-phone thermal camera for early detection purposes. The complete system with a test image is shown in Fig. 2. The use of FLIR ONE camera requires an application which is available for free on Google play store.

#### Image acquisition and measurement procedure

**Fig. 3:**
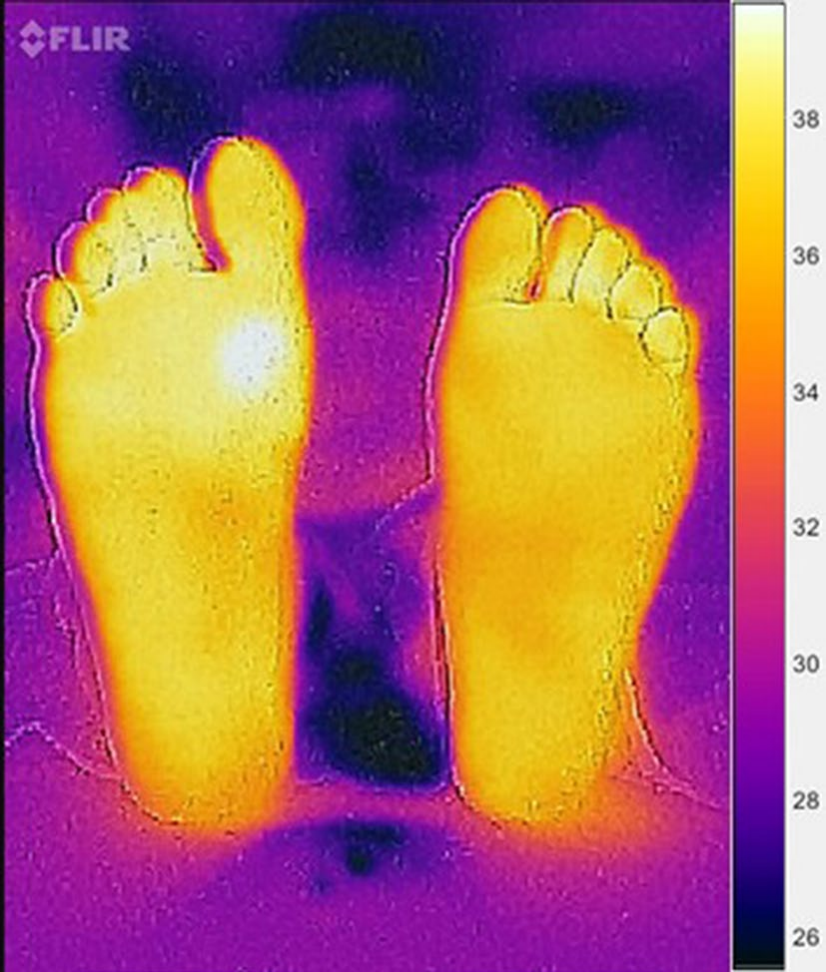
Test image acquired by the camera following the acquisition procedure

The images acquired by FLIR ONE camera were stored in the Smartphone in Joint Photographic Experts Group (JPEG) format. FLIR ONE fused the standard image with the thermal image through Multi Spectral Dynamic Technology (MSX). This was done to provide physical details to the raw thermal image, and therefore, resulted in a better vision as RGB scaled images [20]. In addition, each thermal image was acquired along with a temperature matrix that represents the exact prevalence of temperature within the image. As shown in Fig. 3, a cold towel was held behind the plantar feet to ensure a homogeneous cold background that could be accurately separated from the foreground, which are the plantar feet. Patients are advised to sit and relax for around 3 min in order to maintain stable blood flow within their plantar feet. Furthermore, for accurate analysis, the feet should be placed over a non-reflective surface such as a velvet fabric. Moreover, users are advised to maintain that their feet are located in the center of the field-of-view of the camera for accurate segmentation results. The objects taken to test the algorithms were non-diabetic feet images obtained at room temperature (20–25 °C). To illustrate abnormalities in the feet, we simulated an ulcer by heating coins and materials with different shapes and sizes to a certain temperature. Heating a coin and placing it near the surface of the foot increased the temperature values of the region by around +2.2 °C higher than the normal plantar feet temperature. As previously discussed, Hyperthermia occurs if there is a temperature increase of more than 2.2 °C in some regions within the foot compared to other regions [14, 19].

### Image analysis and interpretation

**Fig. 4:**
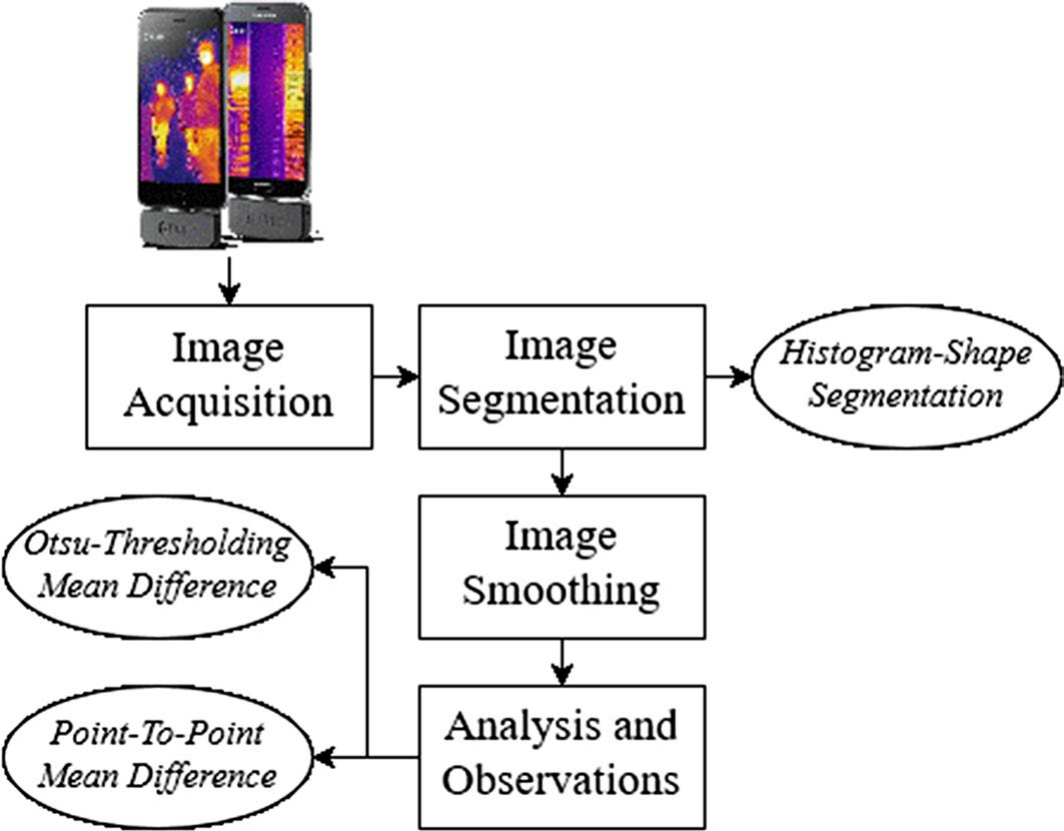
Procedure followed to analyze diabetic feet thermal images

The recorded thermal image was a gray scale image and further analysis and interpretation was conducted. As shown in Fig. 4, the proposed system incorporates various image processing steps including image segmentation, image smoothing, and analysis and observation. The last step was implemented using two techniques: Otsu thresholding mean difference and point-to-point mean difference.

#### Image segmentation

**Fig. 5:**
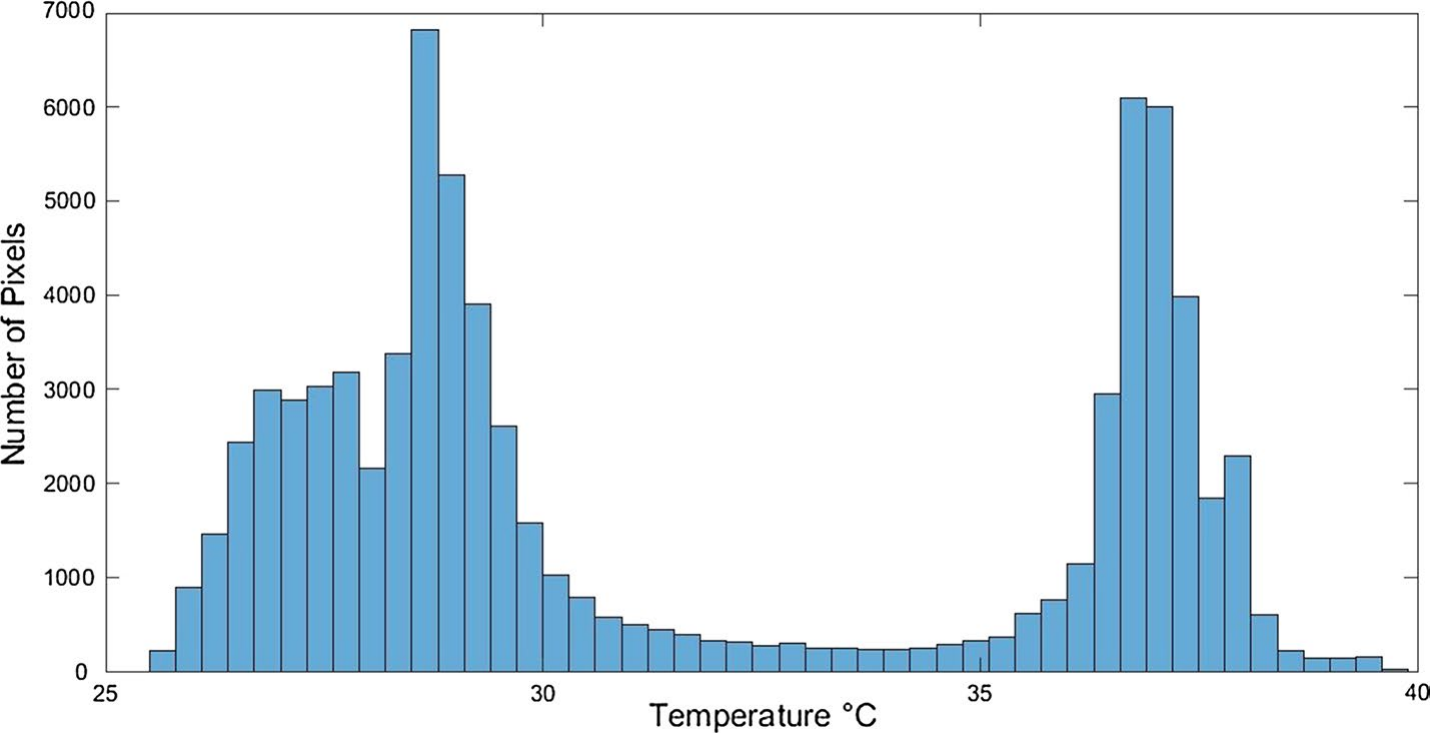
Bi-modal histogram for an acquired test image

The purpose of image segmentation is the extraction of certain objects or segments from original images. In this work, image segmentation was performed twice. The first one was to extract the object of interest from the thermal image, which are the diabetic feet, while the second one was to extract and identify possible ulcer in the diabetic feet. The first segmentation procedure was applied on the raw thermal image with the purpose of extracting the feet from the background, which was in this case the cold towel behind the feet. The technique used for the segmentation process is called Histogram shape thresholding. This technique was applied indicating that thermal images acquired following the acquisition protocol mentioned above resulted in a bi-modal histogram with two peaks, as shown in Fig. 5. The histogram shape thresholding requires finding the optimum threshold to separate the background (dark or cold region) from the foreground (warm object) [22].

In the histogram representation of a sample image shown in Fig. 5, there are two regions that indicate the presence of two different objects; dark and bright objects [22]. The optimum threshold was calculated using Otsu methods [23]. The calculated threshold was used to assign labels for the background (below threshold) and the foreground (above threshold) pixels in the image. Moreover, the probability of gray level *i* is represented by the following equation:

1 $$p_{i} = n_{i} /N$$

where *n*_*i*_ is the number of pixels at level *i* and *N* is the total number of pixels.

Then, the pixels of the images were separated into two regions: R_1_ with gray levels [0, 1,…t] and R_2_ with gray levels [t + 1,…L−1], where *t* is the threshold value. The means of class R_1_ and R_2_ are given by

2 $$u_{1} = \sum\limits_{i = 0}^{t} {ip_{i} /w_{1} }$$

3 $$u_{2} = \sum\limits_{i = t + 1}^{L - 1} {ip_{i} /w_{2} }$$

where *w*_*1*_ and *w*_*2*_ are the gray level probability distributions for the two regions. The total mean of gray levels is given by

4 $$u_{T} = w_{1} u_{1} + w_{2} u_{2}.$$

The within-region variance is given by:

5 $$\sigma_{W}^{2} = \mathop \sum \limits_{k = 1}^{M} W_{k} \sigma_{k}^{2}$$

where $\sigma_{k}^{2}$ is each region variance value and given by:

6 $$\sigma_{1}^{2} = \sum\limits_{k = 0}^{t} {\left( {t - u_{1} } \right)^{2} p_{i} /w_{1} }$$

7 $$\sigma_{2}^{2} = \sum\limits_{k = t + 1}^{L - 1} {\left( {t - u_{2} } \right)^{2} p_{i} /w_{2} }$$

The between-region variance is given by

8 $$\sigma_{B}^{2} = w_{1} \left( {u_{1} - u_{T} } \right)^{2} + w_{2} \left( {u_{2} - u_{T} } \right)^{2}$$

Otsu method picks a threshold *t* by maximizing the between-region variance or minimizing the within-region variance. The total variance, which is the sum of the within-region variance and the between-region variance, is constant for different partitions.

9 $$t = \arg \mathop {\max_{0 \le t \le L - 1} }\limits_{{}} \sigma_{B}^{2} \left( t \right) = \arg \mathop {\min_{0 \le t \le L - 1} }\limits_{{}} \sigma_{W}^{2} \left( t \right)$$

 In MATLAB, the function graythresh automatically find the parameter needed, which is the optimum threshold values *t*, which can separate the two intensity histogram regions. This parameter is given as a normalized value between 0 and 1 of the corresponding scene temperature. The outcome from Otsu thresholding technique was a binary image, with values from 0 to 1, of the segments needed for further analysis, which are the plantar feet.

#### Image smoothing

The technique of Histogram shape segmentation resulted in a binary image of the segmented foreground, which were the plantar feet. However, some images are not easy to segment, especially if there are some parts within the image where the temperature of the background is close to the foreground temperature. As a result, dark objects are marked as bright objects and vise-versa. This affects any further image processing analysis of the plantar feet image. Therefore, image-smoothing techniques were performed to avoid such errors.

At the beginning, borders clearing technique was used to remove any objects that are connected to the border of the image, or even separated in different places within the image. This was done by suppressing any light structures and removing them from the surrounding border of the image. Then, segments’ smoothing was performed to erode the resulting image with a diamond-structuring element. The element used had a single pixel distance from the origin of its structure to the points of the diamond. This prepares the feet segment to be smoothed at the edges and ensures that no unconnected objects are taking place in the image. Finally, the binary segment created might include some interior gaps, therefore, these gaps were filled with hole-filling objects [24]. The plantar feet segments were ready to be analyzed for any occurrence of diabetic feet abnormalities.

#### Analysis and observation

After segmenting the plantar feet image, further processing was performed to identify any possible ulcers or occurrence of hyperthermia (a 2.2 °C difference) [12, 18, 19]. Two techniques were used for this purpose, Otsu thresholding technique (discussed in the previous section) and point-to-point difference technique.

#### Otsu thresholding technique

**Fig. 6:**
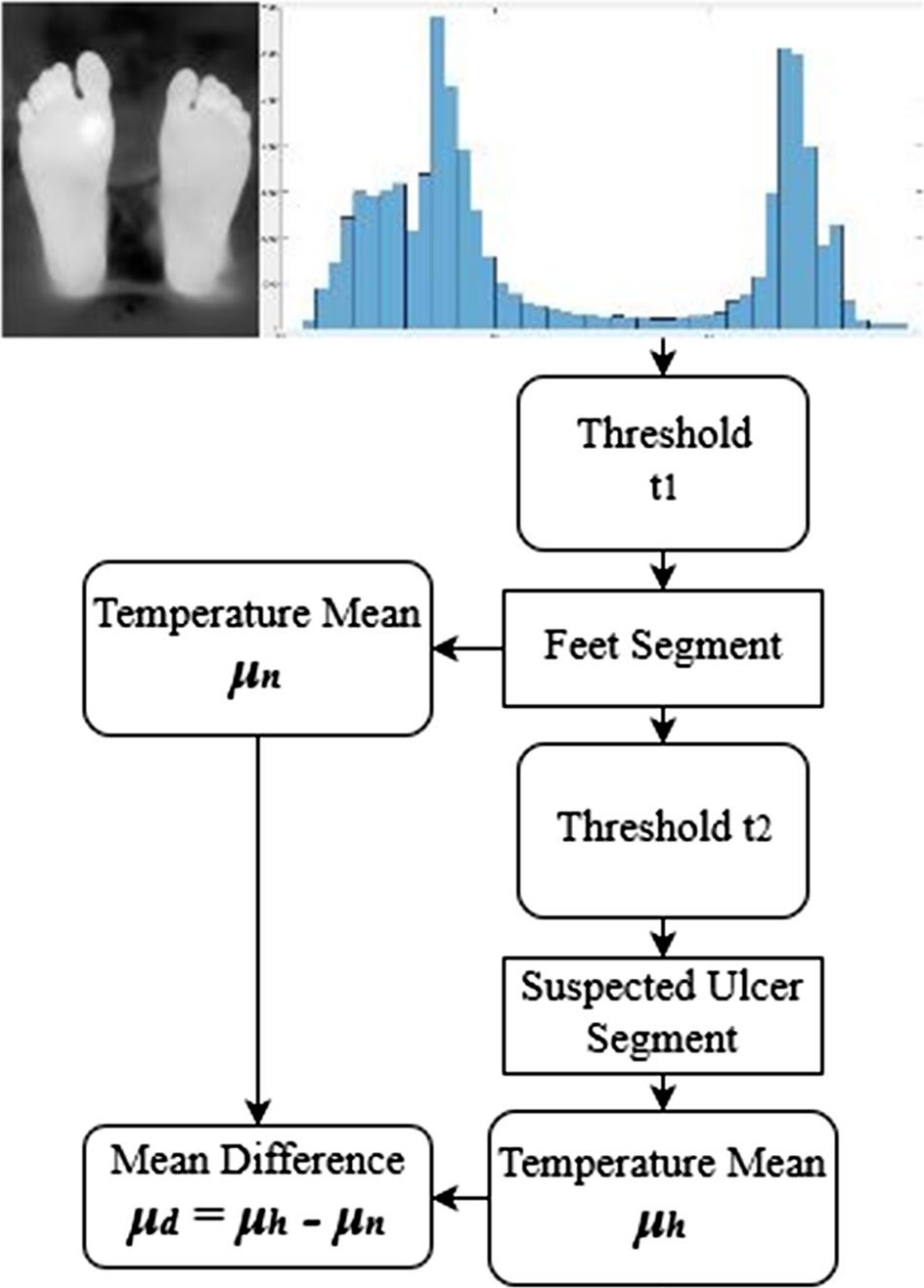
Otsu thresholding mean difference technique procedure

Shown in Fig. 6, this procedure is the same as the thresholding technique applied previously to extract the plantar feet. Here it was performed only on the pixels forming the plantar feet that might have any possible ulceration. Therefore, the feet were now the background and the possible ulcers were the foreground. After thresholding was performed, an independent t test was done to check if the difference between the foreground (suspected ulcer) mean temperature and the background (diabetic feet) mean temperature was greater than 2.2. If the difference was greater than 2.2, then the foreground region was considered as a possible ulcer location.

**Fig. 7:**
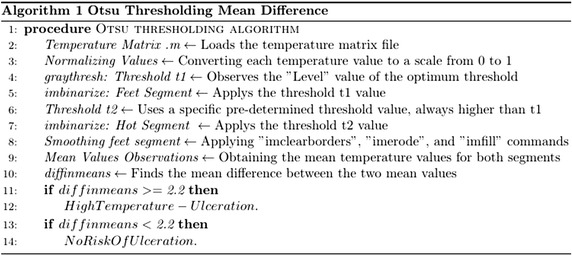
The complete algorithm followed using Otsu thresholding technique

The complete procedure for the proposed algorithm is illustrated on Fig. 7.

#### Point-to-point mean difference

**Fig. 8:**
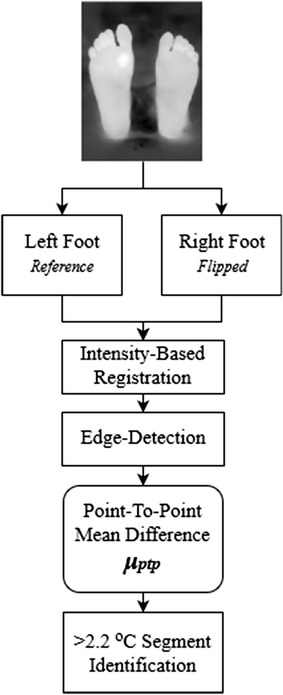
Point-to-point mean difference technique procedure

Another technique was applied for the detection of any possible ulcer, which is called the point-to-point mean difference shown in Fig. 8 [19]. In this technique, the pixel-to-pixel temperature difference between both feet was calculated.

The processed image included both feet; therefore, it was automatically divided into two equal parts; one for the left foot and one for the right foot. As previously mentioned in the acquisition procedure, users are advised to maintain that their feet are in the center of the camera field-of-view for accurate cutting and analysis results. The left foot segment was chosen as the reference foot (the right foot can also be chosen). The deployed technique requires both feet to be aligned together. Hence, two steps were performed; the first one was flipping the right foot to make both feet look identical and the second step was image registration to align both feet together. The adopted image registration technique was the intensity-based registration [25]. Image registration allows both images (left foot and right foot) to be aligned in a way that makes them spatially corresponding to each other [26].

The next step was implementing an edge detection technique to remove the edges from the left and right feet images. The edge detection was applied using the Sobel operators [27]. The resulting image was the difference between the left and the right foot, and pixels with a temperature difference greater than 2.2 °C were identified using thresholding. The resulting image could identify any possible ulcers.

**Table 1:** FLIR ONE complete specifications

Cameras	Lepton and standard VGA
Scene temperature range	−20 to 120 °C
Operating temperature	0 to 35 °C
Sensitivity	0.1 °C
Resolution	160 × 120

**Fig. 9:**
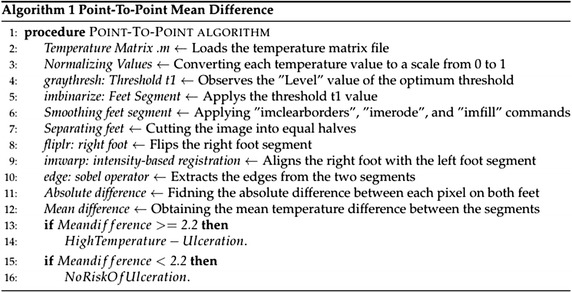
The complete algorithm followed using point-to-point technique

The complete procedure followed in this technique’s algorithm is shown in Fig. 9.

## Results

**Fig. 10:**
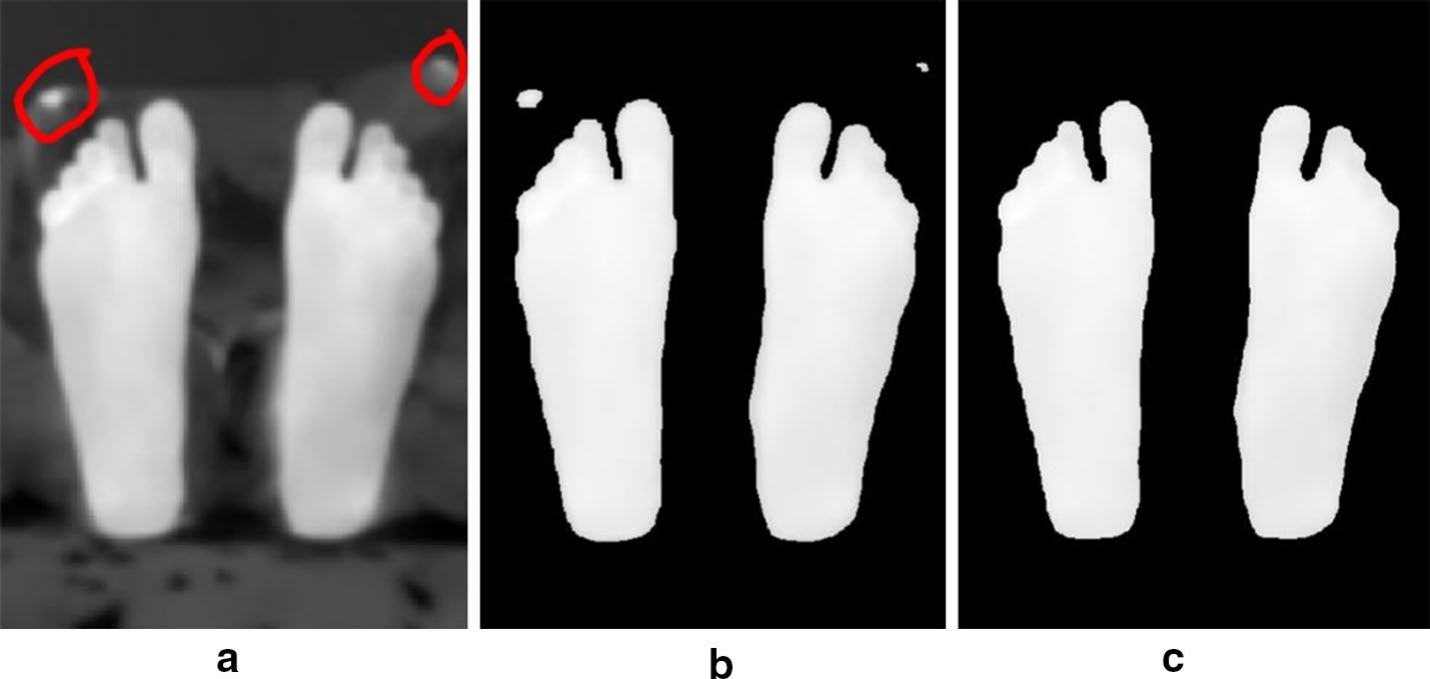
Image smoothing techniques applied on a test image illustrating a noisy object at the background. **a** Acquired Image **b** Segmentation without smoothing. **c** Segmentation after smoothing

The proposed techniques were tested by acquiring thermal images from the feet of a healthy subject. The ulcers were simulated by heating a metal object (a coin) with a diameter of around 1.5 cm. The heated object was placed on the skin to raise its temperature and emulate the possible ulcer (hyperthermia). Eight test images were acquired, one without any skin heating (test image 1) and the seven other images with the feet heated into different locations. The thermal camera temperature measurement was used to make sure that the heated region temperature was more than 2.2 °C higher than the normal feet temperature. A test image illustrating the effect of image smoothing techniques is shown on Fig. 10. A hot object (fingers) is shown in the background, which illustrates an error case in the segmentation and analysis of diabetic foot. Further analysis based on the wrongly segmented plantar feet (Fig. 10b) results in missing the accurate true temperature values for feet and hot regions, therefore, wrong indication of the feet abnormalities situation takes place.

**Fig. 11:**
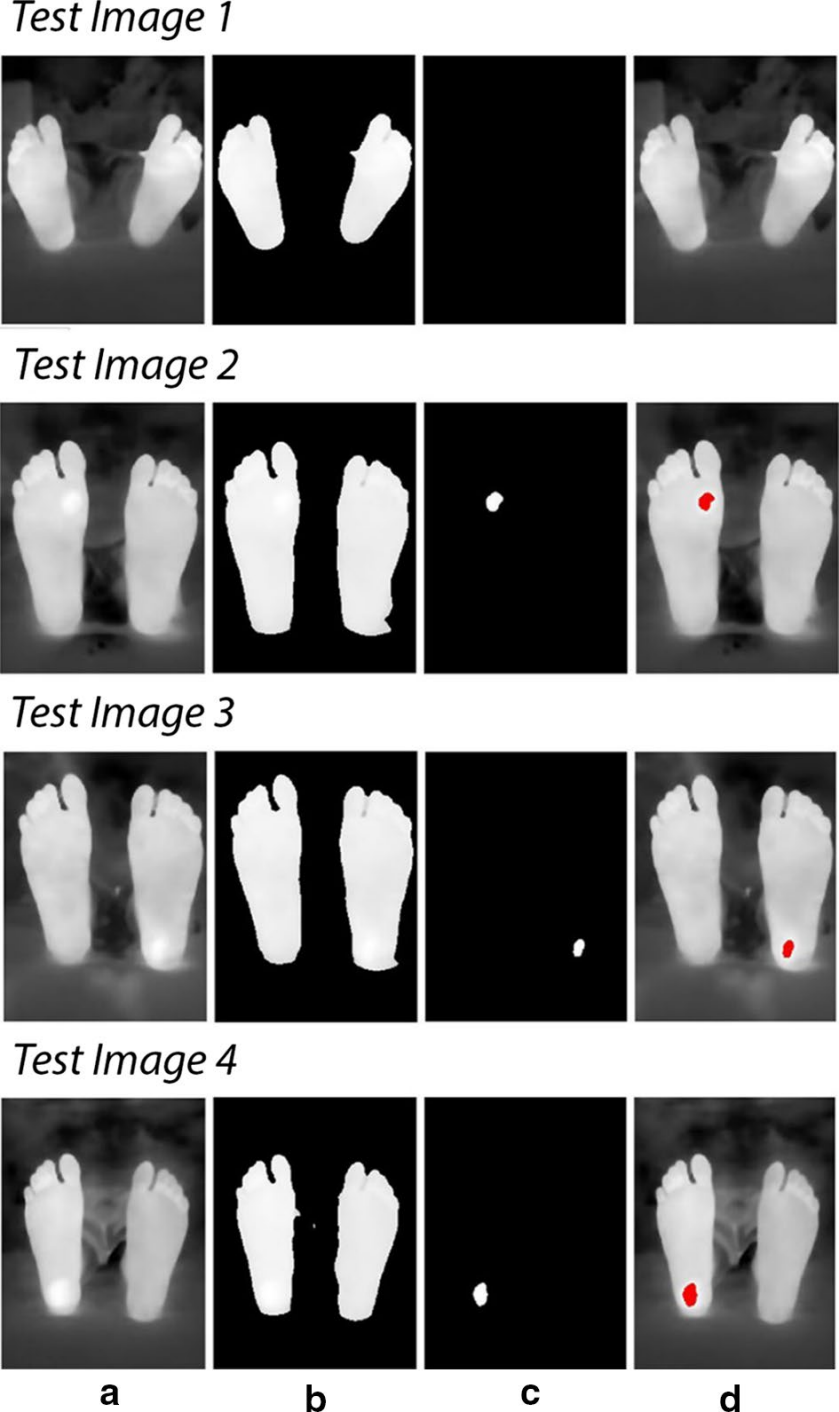
Technique applied on test images 1 to 4. Test image 1 is a healthy feet image. Test images 2 to 4 are images with simulated ulcer. **a** Acquired image, **b** smoothed feet segments, **c** suspected regions, **d** regions on the feet

Figure 11 shows the results from the first four test images, chosen for display, using the Otsu thresholding technique for analysis and observation. Table 2 shows the average temperatures recorded in the identified background (Feet) and foreground (Suspected ulcer) regions in test images 2, 3, and 4. Moreover, the other four test images observations are recorded and mentioned on the table.

**Table 2:** Mean temperature values (µ_n_, µ_h_, and µ_d_) for test images 1 to 8

Observation	Image 1 (°C)	Image 2 (°C)	Image 3 (°C)	Image 4 (°C)	Image 5 (°C)	Image 6 (°C)	Image 7 (°C)	Image 8 (°C)
Feet mean temperature (µ_n_)	33.2	36.9	35.5	35.3	36.2	36.3	36.2	34.8
Suspected region mean temperature (µ_h_)	35.0	39.4	38.0	37.8	38.4	38.5	38.6	37.0
Mean difference (µ_d_)	1.8 (No Ulcer)	2.5	2.5	2.5	2.2	2.2	2.4	2.2

**Fig. 12:**
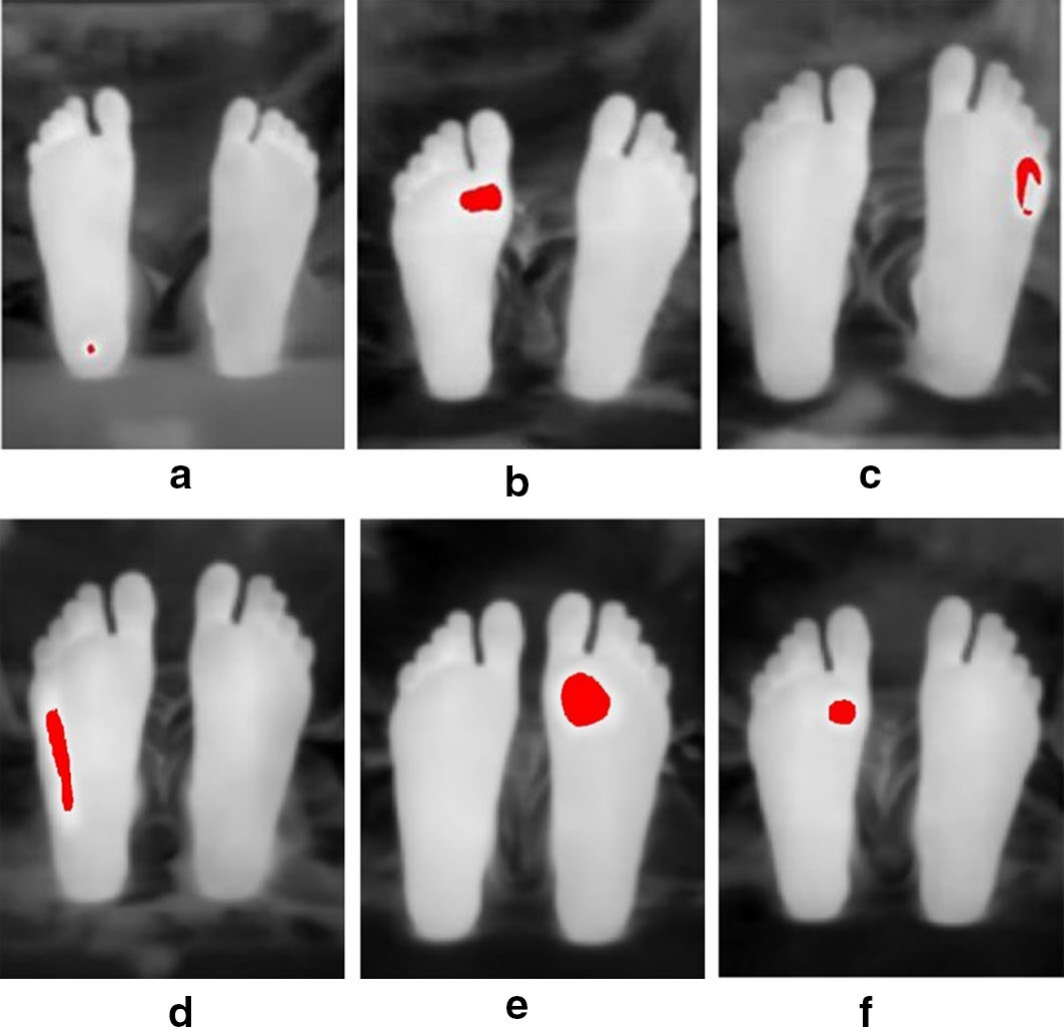
Additional six test images with various shapes to illustrate ulcer. **a** 0.5 cm diameter coin. **b** 2 cm rectangle. **c** Half-shaped ring. **d** 3.5 cm rectangle. **e** 2.5 cm diameter coin. **f** 1 cm diameter coin

Furthermore, the algorithm was tested by adding additional six test images with various shapes simulating ulcer. The shapes used were a small coin of 0.5 cm diameter, a rectangle of 2 cm length, a small half-shaped ring, a 3.5 cm length rectangle, a 2.5 cm diameter coin, and a 1 cm diameter coin. Figure 12 shows the detection results following Otsu thresholding procedure.

**Fig. 13:**
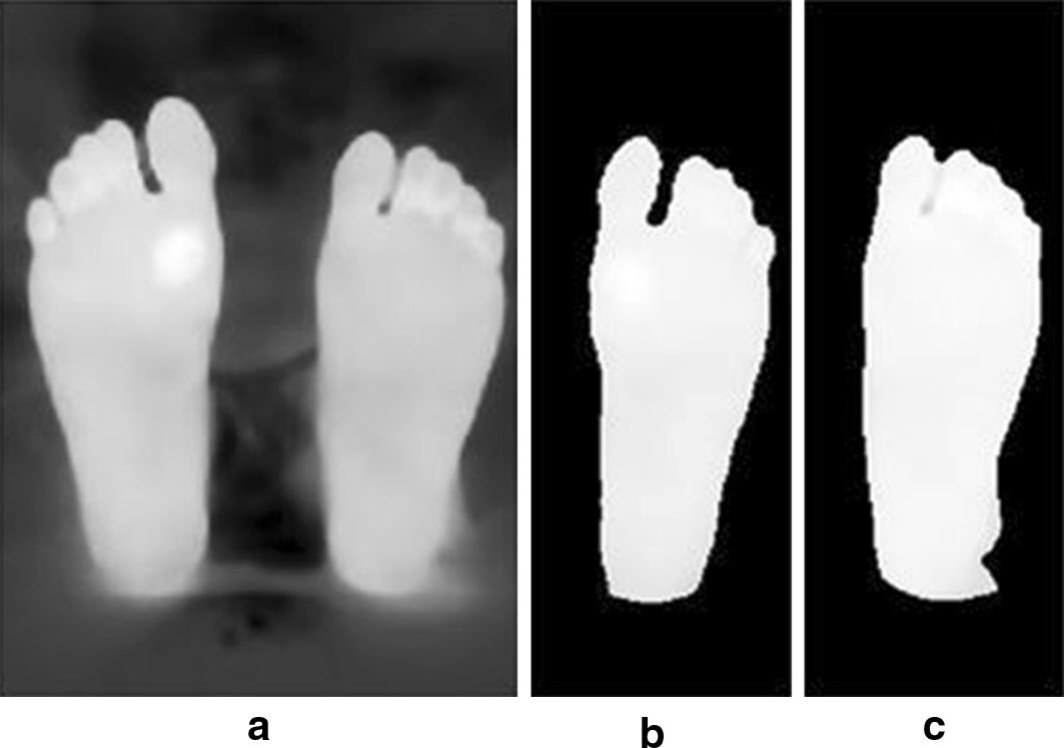
Image flipping and registration applied on testi mage 2. **a** Acquired image. **b** Right foot flipped and registered. **c** Left foot (reference)

**Fig. 14:**
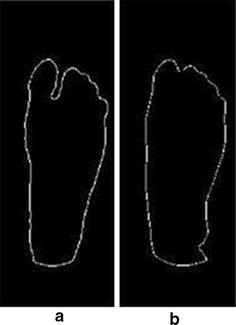
Edge detection technique. **a** Registered right foot contours. **b** Left foot contours

The same test images were used to test the proposed system with the technique of point-to-point difference for image analysis and interpretation. Figure 13 shows an example of the initial steps of this technique which involved image flipping and registration (flipping was done for the right foot). The image splitting and edge detection are shown in Fig. 14.

**Fig. 15:**
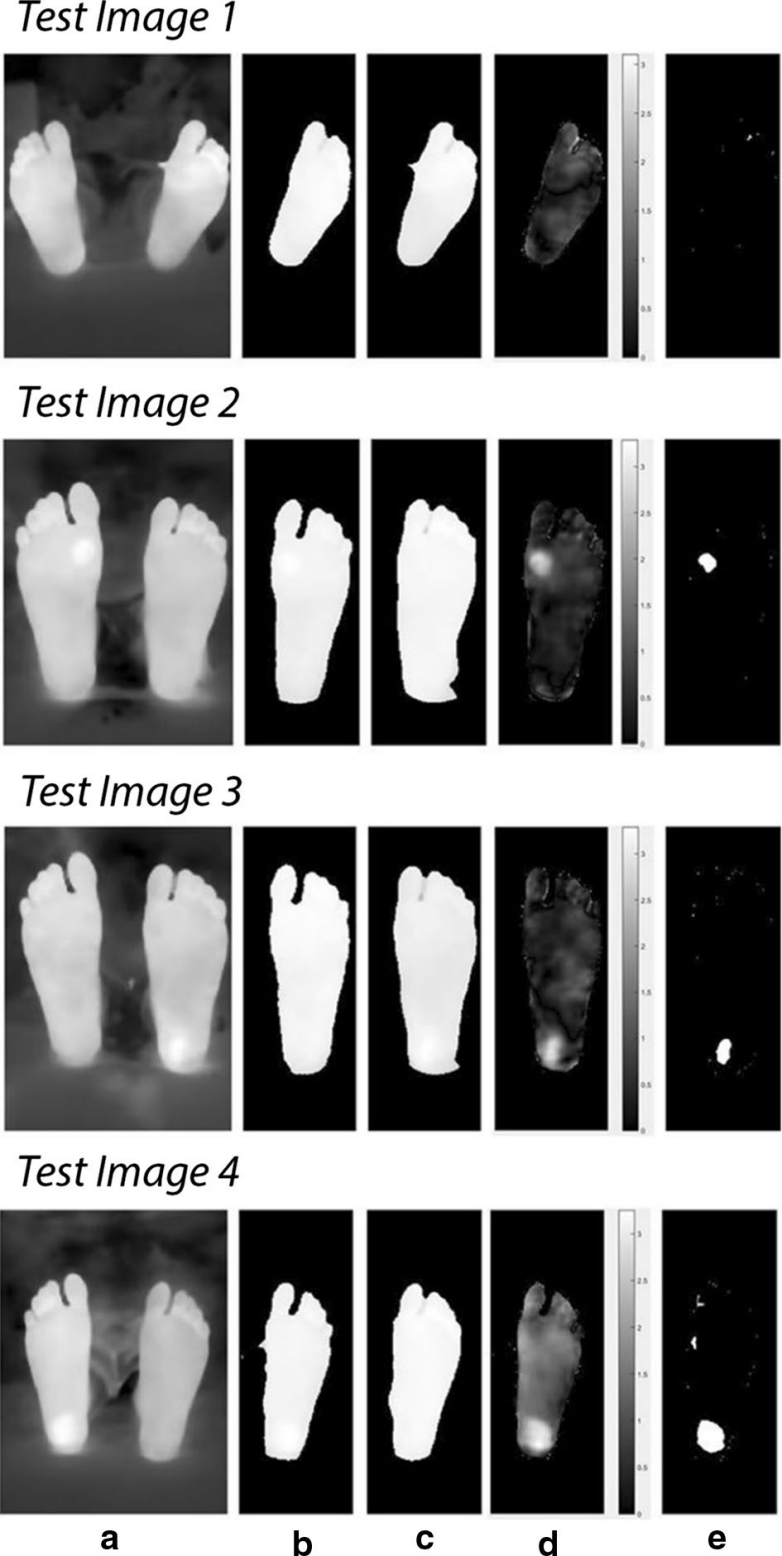
Point-to-point procedure applied on test images 1 to 4. Test image 1 is a healthy feet image. Test images 2 to 4 are images with simulated ulcer. **a** Acquired image. **b** Registered right foot. **c** Left foot (reference). **d** Temperature difference between both feet. **e** Regions of hyperthermia

The results of the point-to-point difference method implemented are shown in Fig. 15 for test images 1 to 4 respectively.

**Fig. 16:**
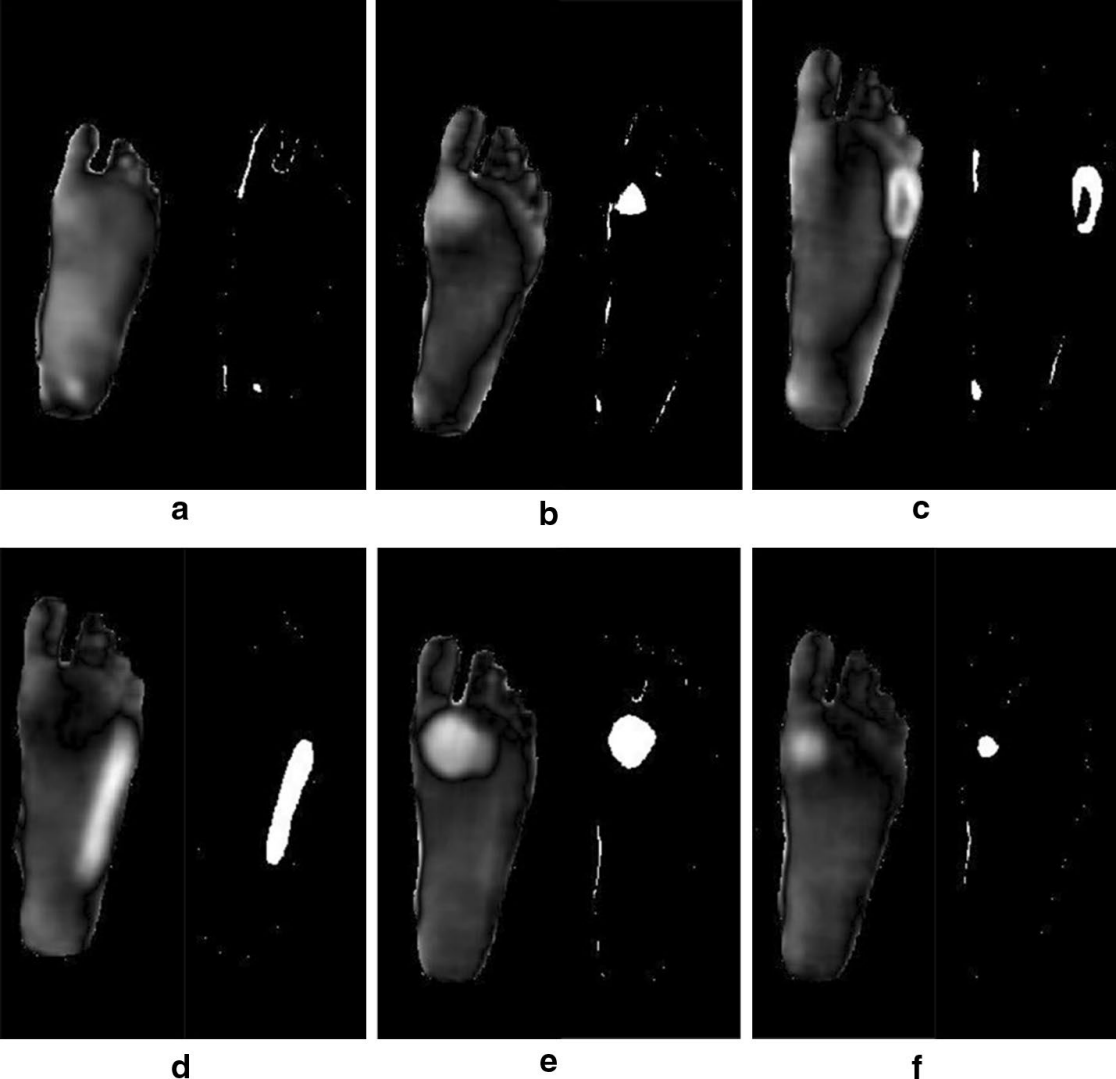
Six test images with different shapes to illustrate ulcer. **a** 0.5 cm diameter coin. **b** 2 cm rectangle. **c** Half-shaped ring. **d** 3.5 cm rectangle **e**. 2.5 cm diameter coin. **f** 1 cm diameter coin

Moreover, the same six test images with the different shapes and sizes were analyzed by the point-to-point algorithm, and the results are shown on Fig. 16.

## Discussion

The main objective of this research was to build a thermal imaging system based on smart phone. The proposed system incorporated the hardware as well as the necessary image processing and interpretation software techniques. According to the obtained results, the proposed system has successfully identified regions with hyperthermia with temperature gradient greater than 2.2 °C which is considered as the value that can be used to identify possible ulcers [14, 19]. The proposed system can also be used to identify temperatures less than this value, but they are not considered as possible ulcers according to the literature. The testing procedure was implemented on four images; one with no temperature gradient introduced and the other three images with thermal gradient at three different locations. The two techniques deployed for image interpretation and analysis were successful in identifying regions with thermal gradient representing possible ulcers. The histogram thresholding techniques used the statistical t-test to verify if the difference between the background and the potential region were statistically different with the mean values listed in Table 2.

The second technique used for analysis and interpretation made use of both legs to be a reference for the background. This technique was used by Vilchuaman et al. [19]. The only difference in this work is that we excluded the edges of the feet using edge detection techniques. This was done because it appeared that the images processed had a high temperature gradient at the edges, which could introduce a kind of false positives. This could be attributed to the processing techniques used, such as image smoothing and image registration. The generated images were for the difference between the two images of the left and right feet as shown in Fig. 14. The average temperature in the difference images generated were 2.4, 2.5, 2.6, 2.2, 2.3, 2.6, and 2.3 °C, for test images 2 to 8, respectively. While in using the Otsu thresholding technique, the average difference between the averages of the background and the foreground were 2.5, 2.5, and 2.5 °C, 2.2, 2.2, 2.4, and 2.2 °C, for test images 2 to 8, respectively. Table 3 shows the complete observations obtained from the actual camera detector, Otsu thresholding, and point-to-point techniques. The actual camera detector values are obtained from the FLIR ONE app direct measurements. Moreover, the six additional test images used illustrated the possibility of detection for various shapes that simulate ulcer. Both algorithms were successful in detecting the shape of the different material used. The current developed techniques do not depend on the shape of ulcer, they depend on the temperature pixels that lies within the suspected region.

**Table 3:** Complete observations for test images 1 to 8

Observations	Image 1 (°C)	Image 2 (°C)	Image 3 (°C)	Image 4 (°C)	Image 5 (°C)	Image 6 (°C)	Image 7(°C)	Image 8 (°C)
Actual camera measured difference	1.7	2.4	2.4	2.5	2.3	2.2	2.5	2.3
Otsu thresholding mean difference	1.8	2.5	2.5	2.5	2.2	2.2	2.4	2.2
Point-to-point mean difference	0	2.4	2.5	2.6	2.2	2.3	2.6	2.3

The entire system was implemented on a Smartphone under MATLAB mobile with processing on a cloud MATLAB server. The use of MATLAB mobile provides the flexibility to do further processing, store the data on a cloud account, and build the necessary interface.

## Conclusion and future work

The proposed system provides a framework to build a complete mobile system that can help diabetic patients self-check their feet for any possible ulcers. The system provides only an indicative tool, not a diagnostic tool, as the final diagnosis should be done by the physician (the gold standard). The future work requires upgrading the system with an advanced thermal camera with higher image quality that can be connected to a mobile in order to perform the necessary processing. Further testing and validation of the system should be performed under clinical environment, which was not possible at this stage due to the strict regulations applied. Moreover, this work can be extended to other possible applications such as wound healing and trauma monitoring.
